# Gene Expression Analysis and Whole Genome Sequencing Reveal the Potential Mechanism of Ciprofloxacin Resistance in a *Salmonella* Dublin Isolate

**DOI:** 10.3390/vetsci13020177

**Published:** 2026-02-10

**Authors:** Kingsley E. Bentum, Amy Leestemaker-Palmer, Stephanie Nuss, Sophia Ballard, Alexandra Montgomery, Woubit Abebe, Temesgen Samuel, Anthony Pokoo-Aikins, Luiz E. Bemudez

**Affiliations:** 1 Center for Food Animal Health, Food Safety and Defense, Department of Pathobiology, College of Veterinary Medicine, Tuskegee University, Tuskegee, AL 36088, USA; 2 Department of Microbiology, Carlton College of Veterinary Medicine, College of Science, Oregon State University, Corvallis, OR 97331, USAstephanie.nuss@oregonstate.edu (S.N.); 3 Oregon Veterinary Diagnostic Laboratory, Bacteriology/Parasitology, Corvallis, OR 97331, USA; 4 Toxicology and Mycotoxin Research Unit, United States Department of Agriculture-Agricultural Research Service (USDA-ARS), U.S. National Poultry Research Center, Athens, GA 30605, USA

**Keywords:** Ciprofloxacin, resistance, *Salmonella enterica* serovar Dublin, quinolone, *gyrA*, mutations, efflux

## Abstract

There is a growing need to understand ciprofloxacin (CIP) resistance in less prevalent *Salmonella* serovars like *Salmonella* Dublin, which causes life-threatening conditions in both humans and animals. This study investigated potential factors contributing to CIP-resistance in a *Salmonella* Dublin isolate. The isolate was detected from an initial screening of 17 biobanked *Salmonella* isolates using the Kirby-Bauer disk diffusion (KBDF) method. The minimum inhibitory concentration (MIC) values of the identified CIP-resistant *Salmonella* Dublin isolate and a CIP-susceptible isolate of the same serovar were also obtained using the broth-dilution (BD) method. The two candidates were then challenged in 1/4 of their respective BD MICs for gene expression analysis, focusing on the *acrAB* efflux genes and the regulator genes *marA*, *ramA*, and *soxS*. Genomes of the isolates were also sequenced using the Oxford Nanopore sequencing platform, and then analyzed for mutations, antimicrobial resistance genes, and plasmids using ABRicate. The SWISS-MODEL server was used for protein modeling and comparison. For our results, the MIC values (KBDF; BD) for the CIP-resistant and CIP-susceptible *Salmonella* Dublin isolates were (1.5 μg/mL; 1.95 μg/mL) and (<0.125 μg/mL; 0.03 μg/mL), respectively. Both isolates had genes (*mdtK*, *emrR*, *emrA*, and *emrB*) notable for fluoroquinolone resistance, with the CIP-susceptible isolate also carrying the IncFII(S) plasmid. Expression of the *acrA*, *acrB*, *ramA*, and *soxS* genes was markedly higher in the CIP-resistant isolate, which also harbored an Asparagine (N) to Serine (S) mutation at position 868 in the GyrA protein. This mutation, however, caused no significant structural change. Despite reporting on a single CIP-resistant *Salmonella* Dublin isolate, our result highlights the potentially significant role of an efficient efflux system in contributing to CIP resistance in this isolate, even when no impactful mutations were identified.

## Simple Summary

Drug resistance in *Salmonella* to important antibiotics such as Ciprofloxacin is becoming an increasing public health threat. Ciprofloxacin resistance is, however, well documented in common *Salmonella* serovars such as *Salmonella* Typhimurium and *Salmonella* Enteritidis, while resistance is reported less frequently in isolates like *Salmonella* Dublin. This study was therefore conducted to investigate potential factors underlying Ciprofloxacin resistance in a *Salmonella* Dublin isolate. This isolate was identified through antibiotic resistance screening of stored *Salmonella* bacteria recovered from various samples, using the Kirby-Bauer disk diffusion method, followed by a broth-dilution method. For comparative analysis, the whole genome of this Ciprofloxacin-resistant *Salmonella* Dublin isolate and another Ciprofloxacin-susceptible isolate from this study cohort were sequenced and screened for resistance genes and plasmids. Also, the two isolates were subjected to gene expression analysis focusing on the efflux genes: *acrAB*, and the regulator genes *marA*, *ramA*, and *soxS*. Finally, protein modeling and genome comparisons were also done to detect mutations in certain genomic segments of interest and their potential impact. Our results showed that the Ciprofloxacin-resistant *Salmonella* Dublin isolate had a very efficient drug efflux activity compared to its Ciprofloxacin-susceptible counterpart. A genetic mutation was also identified in this resistant isolate at the amino acid position 868 of the GyrA protein. However, protein modelling analysis did not show any effective change in structure to suggest a change in function. In summary, although this observation was made in a single Ciprofloxacin-resistant *Salmonella* Dublin isolate, it highlights how an efficient drug efflux activity may contribute to Ciprofloxacin resistance even when no potentially impactful genetic mutations were identified.

## 1. Introduction

Fluoroquinolones (FQs) belong to the second generation of quinolones, and they have enhanced biological activity against microbes [1]. One important member of this class of antibiotics is Ciprofloxacin (CIP), which became clinically available in the mid-1980s [2]. Generally, the enhanced biological activity of FQs allows them to have an extended spectrum against bacteria [3], with drugs like CIP considered the best of treatment options for both invasive and systemic salmonellosis [4].

Sadly, there has been a growing number of quinolone-resistant microbes around the world [3]. Resistance against CIP was practically non-existent when the drug was first introduced [5]. However, the increased use of drugs belonging to the FQ class of antibiotics, especially in livestock production, has greatly contributed to a corresponding high incidence of resistant species against CIP among important pathogens like *Salmonella* [6]. A typical example was the rise in the proportion of FQ-resistant *Salmonella* Typhimurium from 1% in 1994 to 12% in 1996 after the drug’s approval for use in veterinary medicine in the U.K. in 1993 [7]. Often, these antibiotics are used among many livestock establishments at sub-inhibitory concentrations for disease prevention or as feed additives [5,8]. This practice has undoubtedly contributed to the development of resistant strains with the potential to infect both humans and animals [9].

Because all FQs have the same mechanism of action, it is easy for resistance to one member of the drug class to be conferred to another [7]. For instance, resistance against enrofloxacin, which is intended for animal use, can translate to resistance against CIP, which is primarily intended for human use. In addition, both drugs are members of the second generation of FQs [1]. The mechanism of action of quinolone drugs against microbial organisms is the inhibition of bacterial DNA replication by blocking the activity of the DNA gyrase and DNA topoisomerase IV enzymes [3]. The *gyrA* and *gyrB* genes code for the DNA *gyrase* enzyme, while the *parC* and *parE* genes code for the topoisomerase IV enzyme [3]. Hence, any alterations in the drug targets in these target enzymes within the pathogen can potentially confer resistance against the drug [2].

Since the first report of a CIP-resistant *Salmonella* in 1990, many other resistant isolates have been identified [10,11,12]. However, compared to the other members of *Enterobacteriaceae*, CIP-resistance, and for that matter, quinolone-resistance in *Salmonella* is considered to be an evolving phenomenon [13]. Furthermore, studies discussing quinolone-resistance in less frequent *Salmonella* isolates, such as *Salmonella* Dublin, are quite scarce, making it more difficult to assess their public health risk better [14]. This is partly because it is quite uncommon to isolate CIP-resistant *Salmonella* Dublin. For instance, in a recent study using a global dataset of 1303 *Salmonella* Dublin genomes, only one isolate was identified to be carrying genetic mutations associated with CIP resistance [15]. Another study involving 74 *Salmonella* Dublin isolates from ten federal states in Germany reported no CIP resistance except for one strain [16]. Finally, a large collection of *Salmonella* Dublin isolates collected over decades in Japan had none of the isolates showing resistance to fluoroquinolones (including CIP) [17].

Considering the high morbidity and mortality rates associated with *Salmonella* Dublin infection [18], and given the potential public health implications of CIP-resistant *Salmonella* Dublin, this study was conducted to investigate how chromosomal mutations, plasmids, antimicrobial resistance genes, and efflux pumps interplay to contribute to CIP resistance in an identified *Salmonella* Dublin isolate.

## 2. Materials and Methods

### 2.1. Selection of Isolates and Bacterial Culture

A total of 17 *Salmonella* isolates, biobanked by the Biomedical Research Laboratory at Oregon State University, were selected on 25 June 2023, for initial antibiotic susceptibility screening. The associated metadata for the isolates is presented in Table 1. The isolates were removed from a −80 °C freezer (Thermo Scientific, Asheville, NC, USA). Using a sterile inoculating loop, flakes of the stock cultures of the isolates were then plated on prepared MacConkey agar (BD, Franklin Lakes, NJ, USA) plates. The plates were then incubated at 37 °C for 24 h. Colonies from the stock cultures were used for downstream analysis.

### 2.2. Antimicrobial Susceptibility Testing

Tryptic Soy Agar culture slants of the 17 isolates were prepared from the revived colonies and sent to the Oregon Veterinary Diagnostic Laboratory on 7 July 2023, for antimicrobial susceptibility testing. All the isolates were tested for their susceptibility to CIP (5 µg) using the Kirby-Bauer disk diffusion method. Disk diffusion zones were measured using the BIOMIC V3 Reader (Giles Scientific, Santa Barbara, CA, USA). For subsequent bacterial challenge with CIP, the susceptibilities of the isolates were also analyzed using the broth dilution method to obtain the minimum inhibitory concentrations (MICs) to determine the appropriate drug exposure concentrations. For both disk diffusion and broth dilution methods, the susceptibility categories and the MIC of the isolates were interpreted according to the established criteria of the Clinical and Laboratory Standards Institute (CLSI) [19].

### 2.3. Bacteria Challenge with Ciprofloxacin

From the disk diffusion method, CIP-resistant and CIP-susceptible isolates belonging to the same serovar were selected for antibiotic challenge for comparative analysis. The MICs of the selected candidates to CIP were obtained using the broth dilution method, and to also determine the appropriate CIP challenge concentrations for the gene expression analysis. The isolates were tested against various concentrations of CIP (Millipore Sigma, Burlington, MA, USA) prepared using Mueller–Hinton broth (Becton, Dickinson and Company, Franklin Lakes, NJ, USA). Briefly, 900 µL each of CIP concentrations ranging from 250 µg/mL to 0.01 µg/mL, obtained by 2-fold serial dilutions from the highest to the lowest concentration, were prepared. Fresh bacterial cultures grown in Mueller–Hinton broth were centrifuged at 2000 rpm for 10 min. The supernatant was discarded, and the pellet was resuspended in 5 mL of HBSS 1X solution (Gibco Laboratories, Grand Island, NY, USA) to obtain a bacterial stock culture. From the stock culture, a 0.5 McFarland concentration of bacterial culture was prepared using HBSS 1X solution, and 100 μL each of this bacterial culture was inoculated into the various CIP concentrations. The inoculated samples were then incubated for 24 h at 37 °C to determine the broth dilution MICs of our isolates. The next day, to induce a CIP exposure response, rather than a CIP-induced cell death, and to eliminate the possibility of stationary-phase RNA remodeling, 1 mL of freshly prepared 0.5 McFarland concentration of each of the CIP-resistant and CIP-susceptible isolates was inoculated into 9 mL of 1/4 their respective broth dilution MICs for CIP (Supplementary Figure S1). A control experiment was also set up with no CIP supplementation of the media. Both the experimental and control cultures were incubated at 37 °C for 10 h.

### 2.4. RNA Extraction and Gene Expression Analyses Targeting Efflux Transporter Genes and Regulators

The expression of the *acrAB* efflux transporter genes and the regulator genes *marA*, *ramA*, and *soxS* was measured in our analysis. Following the incubation of the CIP-challenged and control cultures, the cultures were centrifuged at 14,000 rpm for 10 min, and the supernatant was discarded. Total RNA was then extracted from the culture pellet using the Zymo RNA/DNA Miniprep Kit (Zymo Research, Tustin, CA, USA) according to the manufacturer’s instructions. Residual genomic DNA was removed from the extracted RNA using DNase I Roche (Millipore Sigma, Burlington, MA, USA) following the manufacturer’s protocol. For gene expression analysis, the extracted RNA was first reverse transcribed using the iScript reverse transcription kit (Bio-Rad Laboratories, Hercules, CA, USA) to produce cDNA, which was used as a template for real-time PCR using the qRT–PCR iQ™ SYBR^®^ Green Supermix (Bio-Rad Laboratories, Hercules, CA, USA) according to the manufacturer’s protocol. The qRT–PCR set-ups were conducted in triplicate, and the expression level of each gene was calculated using the average of the three independent set-ups. The primers used for the qRT–PCR are listed in Table 2. The following cycling conditions: an initial denaturation at 95 °C for 3 min, followed by 40 cycles of denaturation and annealing at 95 °C for 10 s and 60 °C for 30 s, respectively, were used as recommended by the manufacturer. Finally, the relative quantities of each expressed gene transcript were measured based on their cycle threshold (CT) values, and the expression of the genes of interest by both treated and untreated cultures was analyzed using the 2^−ΔΔCT^ method [20]. Fold change results were only reported descriptively (with no statistical analysis done) due to prior averaging of the CT values and the small sample size involved.

### 2.5. Whole Genome Sequencing of CIP-Resistant and CIP-Susceptible Salmonella Isolates

Both the CIP-resistant and CIP-susceptible isolates were freshly cultured on MacConkey agar plates, and DNA was extracted from the cultures for whole-genome sequencing. DNA extraction was done using the Zymo Quick-DNA High Molecular Weight Kit with Magnetic Beads (Zymo Research, Irvine, CA, USA) using modifications for homogenization and lysing, then continued with standard protocols. Careful consideration was taken to reduce mechanical shearing, including minimizing pipetting, to reduce fragmentation as much as possible. Modifications included mixing approximately 10^9^ total bacteria cells in 400 µL of Zymo DNA/RNA Shield buffer and incubating at 37 °C for 4 h, then adding 20 µL of Proteinase K and incubating for an additional hour. The ‘Cultured Bacterial and Fungal Cells’ protocol was then followed from step 4 as listed. Heatless speed-vacuuming was used for 1 h to raise the concentrations of the isolate’s DNA after extraction. Following extraction, genomic DNA libraries were prepared using the Oxford Nanopore Rapid Sequencing Barcoding kit (SQK-RBK-114-24) at 200 ng total input. The respective genomic DNA of the serovars was sequenced using the Oxford Nanopore MinION Mk1D long-read sequencer using a R10.4.1 flow cell and processed using the MinKNOW v5.2.3, with live basecalling performed with the ‘Super-Accurate Basecalling’ model using Dorado with a minimum quality score of 9 (Oxford Nanopore Technologies, Oxford, UK). The raw passing reads were then analyzed with Oxford Nanopore’s Epi2ME v5.2.5 software, using the wf-bacterial-genomes workflow v1.4.2. Generated assembled contigs from the workflow were used for further downstream investigations. Raw reads from both isolates have been deposited in the National Center for Biotechnology Information (NCBI) under the bioproject accession PRJNA1369519.

### 2.6. Plasmid, Antimicrobial Resistance, and Mutation Analysis

Using ABRicate (version 1.0.1) with a coverage threshold of 95% and an identity threshold of 80%, the resulting assembled contigs were screened against the Comprehensive Antibiotic Resistance Database (CARD) and the PlasmidFinder database, both updated on 17 October 2025. The two databases were used for antimicrobial resistance gene and plasmid detection, respectively. Also, a customized database built from the *gyrA*, *gyrB*, *parC*, and *parE* genes of the reference isolate—*Salmonella enterica* subsp. enterica serovar Dublin str. CT_02021853 (NCBI accession NC_011205.1) was constructed, and the assembled contigs were screened against the customized database for the detection of those genes in our isolates’ assembled contigs. For each of the two isolates, contigs that reported hits for each gene target were aligned to the reference gene using Molecular Evolution Genetics Analysis (MEGA—version 12) to detect nucleotide mutations. Furthermore, the ExPASy (**Ex**pert **P**rotein **A**nalysis **Sy**stem) translate tool (https://web.expasy.org/translate/), accessed on 10 November 2025, was used to convert the nucleotide sequences into their corresponding amino acid sequences and again aligned to the reference sequence to detect mutations at the amino acid level.

### 2.7. Protein Modelling

Amino acid sequences were modelled into proteins using the SWISS-MODEL automated protein structure homology-modelling server (https://swissmodel.expasy.org/), accessed on 21 October 2025. The generated protein structures were superimposed and compared in a 3-D format to analyze the effect of mutation on the general structure of the modelled proteins.

## 3. Results

### 3.1. Antimicrobial Susceptibility Testing of Isolates Against Ciprofloxacin

Out of the 17 isolates screened, only one isolate, identified as *Salmonella* Dublin recovered in 2023 from the fetal stomach content of a bovine, was resistant to CIP. The MIC value for this resistant isolate, as determined by the Kirby-Bauer disk diffusion method, was 1.5 μg/mL, as shown in Table 3. For this CIP-resistant isolate and its CIP-susceptible counterpart, the MIC values obtained through the broth dilution method were 1.95 μg/mL and 0.03 μg/mL, respectively (Table 3).

### 3.2. Gene Expression Analysis for the CIP-Resistant and CIP-Susceptible Salmonella Dublin Isolates

**Figure 1:**
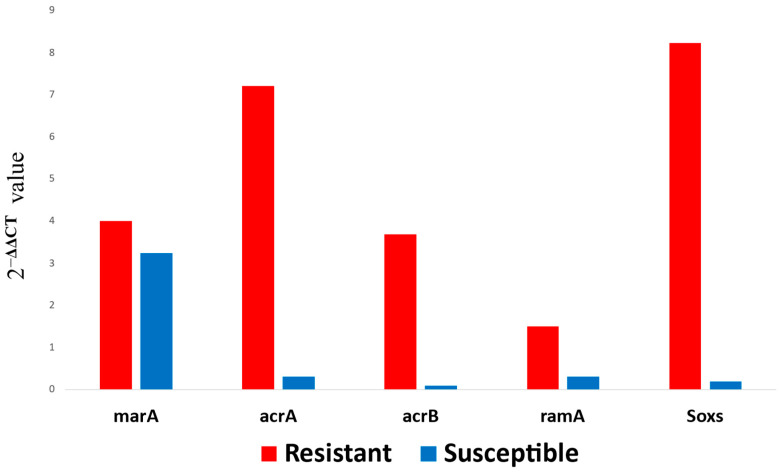
**Bar charts showing results for Fold-change** ***analysis* using the 2^−ΔΔCT^ method**. The different expression levels of efflux transporter genes *acrAB,* and regulator genes *marA*, *ramA*, and *soxS* are shown, where red bars represent gene expression levels for the CIP-resistant isolate, while blue bars represent gene expression levels for the CIP-susceptible isolate.

It was observed that, although the expression of the *marA* gene was almost at equal proportions for both the resistant and the susceptible isolate, the expression levels for the *acrA*, *acrB*, and the *soxS* genes were markedly higher in the CIP-resistant isolate compared to the CIP-susceptible isolate (Figure 1).

### 3.3. Chromosomal Mutations Identified in the Isolates

**Figure 2:**
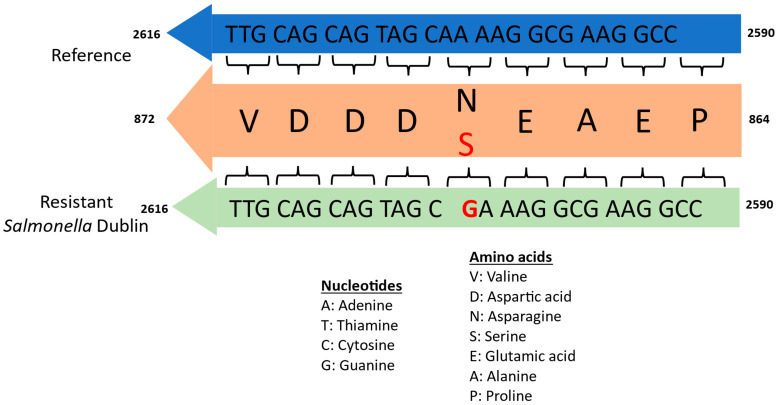
**Sequence alignment showing the mutation site in the** ***gyrA* gene resulting in a codon switch.** A representation of an alignment segment of the *gyrA* gene of the reference isolate: *Salmonella* enterica subsp. enterica serovar Dublin str. CT_02021853 (NCBI accession NC_011205.1) and the CIP-resistant *Salmonella* Dublin. All nucleotides are grouped in codons and shown on the respective segments: blue for the reference isolate and green for the CIP-resistant isolate. The consensus amino acid segment is also shown in orange and placed between the nucleotide segments, with each amino acid corresponding to its respective codons as shown by curly brackets. Both nucleotides and amino acids are shown in their single-letter codes, where red denotes mutation. A legend for interpreting these codes is shown below the segments.

No mutations were identified in the CIP-susceptible isolate. However, the gyrase genes (*gyrA* and *gyrB*) and the topoisomerase genes (*parC* and *parE*) of the CIP-resistant isolate showed several synonymous mutations at the nucleotide level, as shown in Supplementary Table S1. The only non-synonymous mutation occurred as a switch from Adenine (A) to Guanine (G) at position 2603, resulting in a corresponding amino acid codon switch from Asparagine (N) to Serine (S) at position 868 (Figure 2).

### 3.4. Protein Modeling

**Figure 3:**
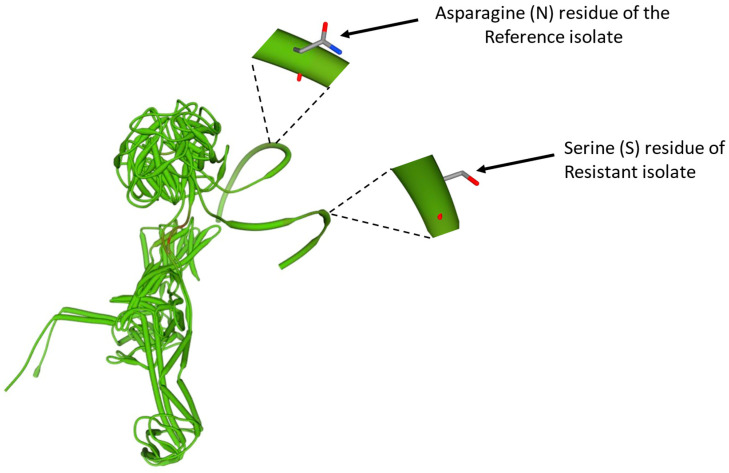
**Superimposed GyrA protein models from reference and CIP-resistant isolates.** The superimposed GyrA protein models of both the reference strain *Salmonella enterica* subsp. enterica serovar Dublin str. CT_02021853 (NCBI accession NC_011205.1) and the CIP-resistant *Salmonella* Dublin isolate in this study. The relative position and structure of the Asparagine (N) amino acid residue in the reference strain and its substitution by a Serine (S) amino acid residue in the resistant strain are shown by arrows, respectively.

The GyrA protein sequences of both the resistant isolate and reference isolate were modeled using the DNA gyrase subunit A of *Salmonella* as a template, with a sequence identity of 100 and 99.89, respectively. The structures generated for the two proteins were very comparable except for the structure of the single amino acid switch, as shown in Figure 3.

### 3.5. Plasmid and Antimicrobial Resistance Genes

For resistance genes, both the CIP-resistant and CIP-susceptible isolates carried the *mdtK*, *emrR*, *emrA*, and *emrB*, which have previously been noted to have activity against fluoroquinolones (Supplementary Data: Tables S2 and S3). No plasmids were detected in the CIP-resistant isolate. However, the IncFII(S) plasmid was identified in the CIP-susceptible isolate (Supplementary Data: Tables S4 and S5).

## 4. Discussion

This study adds to our understanding of CIP resistance in *Salmonella* Dublin, which has, over the years, been an underexplored topic. Although it focuses on the less-frequent finding of a single CIP-resistant isolate, the robust combination of two different susceptibility testing, gene expression analysis, whole-genome sequencing, and protein modeling provides a wide-view approach to understanding the potential origin of CIP resistance in this single *Salmonella* Dublin isolate.

The continuous decrease in the susceptibility of *Salmonella* Dublin to drugs like CIP has been a growing concern over the years [24]. However, compared to other highly prevalent serovars such as *Salmonella* Enteritidis and *Salmonella* Typhimurium, few studies exist investigating this phenomenon because CIP-resistant *Salmonella* Dublin is less frequently isolated overall [14]. This, undoubtedly, has impeded progress in knowing the repertoire of mechanisms *Salmonella* Dublin adopts in conferring resistance against CIP, including key genetic mutations. In providing a comprehensive summary of reported mutations in the quinolone resistance-determining region (QRDR) of quinolone-resistant *Salmonella* isolates, Shaheen et al. noted that continuous research may lead to the expansion of these regions [14]. In addition, the frequency and distribution of these mutations may not necessarily be a *Salmonella* serovar-specific phenomenon [25].

Although our CIP-resistant *Salmonella* Dublin isolate carried a GyrA Asp868Ser mutation, this mutation was outside the defined QRDR for *Salmonella* [14]. In *Salmonella*, some commonly identified mutations in the GyrA protein have included Ser83Phe, Arg47Ser, Asp82Asn, Asp87Asn/Gly/Tyr/Val, Glu133Gly, and Asp147Gly [8,14,26,27]. Considering the GyrA QRDR, which spans approximately amino acid positions 67 to 106 [14], these notable mutations: Arg47Ser, Glu133Gly, and Asp147Gly, just like the one identified in this study, are clearly outside the QRDR, further supporting the suggestion that the QRDR could be potentially expanded, especially where the mutations are confirmed to confer resistance. We hypothesize that our identified mutation may not have any significant impact on the function of the GyrA protein and perhaps may not have contributed to the observed CIP-resistant phenotype of our isolate. This is mainly because no significant change in the structure of the GyrA protein was observed. This claim about the non-functionality of our observed mutation, however, is a subject we intend to investigate further in future studies. It is also important to note that not all quinolone-resistant *Salmonella* isolates harbor mutations in the *gyrA* gene, and resistance may not always correlate with such mutations [28,29].

In this study, it was also observed that both the CIP-resistant and CIP-susceptible isolates carried the multidrug-resistant (MDR) genes *mdtK*, *emrR*, *emrA*, and *emrB*, which act against notable drugs like FQs. Nevertheless, an active expression of multi-drug efflux-pump-related genes in the CIP-resistant isolate in this study may have informed the observed resistance even in the absence of a potentially non-impactful mutation. In fact, upregulation of MDR efflux genes is key in mediating quinolone resistance [8]. The importance of these efflux pumps to producing resistant isolates cannot be overemphasized. This is because only a target site mutation in the all-important *gyrA* gene may not necessarily translate to clinical resistance if no efflux pump activities exist [30]. This underscores the significant synergy of both mutations and efflux activity in producing drug resistance in microbes.

As earlier stated in previous research, the action of multidrug efflux transporters in extruding several chemotherapeutic agents greatly helps in conferring resistance [31]. The overexpression of *acrA* and *acrB* genes has been strongly correlated with FQ and MDR in *Salmonella* and other pathogens [12,32]. Hence, the well-expressed *acrAB* genes in the CIP-resistant isolate in this study suggest their potential significance in contributing to the observed resistant phenotype. Furthermore, the *acrAB* in *Salmonella* is part of the *acrAB-tolC* multidrug efflux system, which is also mainly regulated by the *ramRA* locus [21]. The *ramA* gene is responsible for the activation of the transcription of *acrAB* and *tolC* genes, and the gene is, in turn, repressed by the *ramR* gene [21]. The *ramA* gene, together with the *marA* and the *soxS* genes, components of the mar box operon [8], are essential in drug protective responses as their upregulation increases efflux activity for cellular detoxification [30,33]. This detoxification is ultimately achieved through the activation of *acrAB* transcription [8].

The *ram*A gene was, however, the least expressed in the CIP-resistant isolate, and this may have been due to expression at levels just enough to sustain drug efflux activity. This is because a high expression of the *ramA* gene activates a feedback reaction from the *ramR* gene to repress its activity [34]. The *ramA* gene activates the acrAB efflux pump. Hence, its inhibition by *ramR* due to overexpression will lead to a counter-survival action. On the other hand, the *soxS* gene was among the least expressed genes in the CIP-susceptible isolate, and this may have contributed to its susceptible phenotype. The superoxide (*soxS)* gene, as the name suggests, is upregulated whenever bacteria are under a threat of oxidative stress, as it is essential in initiating the transcription of genes aimed at reducing superoxide and nitric oxide stress in the bacteria [30]. CIP induces the production of superoxide anion and other reactive oxygen species in microbes [35], and a reduced ability to extrude these from the bacterial cell can compromise survival. Despite the cogent inferences made regarding our comparative gene expression results for these genes, future studies aim to adopt protein quantification and functional inhibition assays to elucidate these observations.

Finally, the mechanisms of quinolone resistance in many bacterial pathogens can be summed up as: (i) mutations in the QRDRs of the *gyr* and *par* genes on the chromosome leading to low-affinity binding of the topoisomerase enzymes; (ii) plasmid-mediated quinolone resistance (PMQR), where pathogens acquire the *qnr*, the *aac(6′)-lb-cr*, or *oqxAB* and *qepA* genes, thus decreasing FQ activity and increasing their efflux; and (iii) the downregulation and upregulation of multidrug efflux pumps to ultimately reduce cellular concentrations of FQs [3,4,36,37]. Although many CIP-resistant *Salmonella* rely on plasmid-mediated quinolone resistance (PMQR) [6], no plasmid was detected in our CIP-resistant isolate.

This study was limited by the analysis of only one identified CIP-resistant isolate among the cohort of *Salmonella* isolates screened. Hence, all findings and discussions were made based on this isolate, limiting the generalization of our findings to the entire serovar. Nevertheless, the strength of this study lies in reporting the rare isolation of a CIP-resistant *Salmonella* Dublin isolate and adopting a combination of robust analyses to investigate the observed resistant phenotype. Our result highlights the potentially significant role of an efficient efflux system in contributing to CIP resistance in this *Salmonella* Dublin isolate, even when no impactful mutations were identified.

## Figures and Tables

**Table 1:** **Metadata of biobanked** ***Salmonella* isolates.**

Number	Accession	Organism	Species	Source	Date Obtained
1	85-1235	*Salmonella* Dublin	n/a	n/a	1985
2	02-742	*Salmonella* Typhimurium	n/a	n/a	2002
3	09-10043	*Salmonella* Enteritidis	Equine	n/a	2009
4	13-427	*Salmonella* Typhimurium	n/a	n/a	2013
5	15-3130	*Salmonella* Typhimurium	n/a	n/a	2015
6	20-04335	*Salmonella* Typhimurium	Equine	n/a	2020
7	21-02381	*Salmonella* Typhimurium	Avian	n/a	2021
8	21-02399-B	*Salmonella* Typhimurium	Avian	n/a	2021
9	15V01330	*Salmonella* Enteritidis	Avian	n/a	2015
10	C202670019	*Salmonella* Typhimurium	Equine	Feces	2020
11	C210050040	*Salmonella* Typhimurium	Avian (Pine siskin)	Liver/Small Intestine	2021
12	C210080050A	*Salmonella* Typhimurium	Bovine	Large intestine contents	2021
13	C210460048-1	*Salmonella* Typhimurium	Canine (domestic)	Draining tract	2021
14	C220050014	*Salmonella* Typhimurium	Bovine	Feces	2022
15	C2301000070-2	*Salmonella* Typhimurium	Caprine	Uterus swab	2023
16	C230200031	*Salmonella* Typhimurium	Bovine	Liver	2023
17	C230310056	*Salmonella* Dublin	Bovine	Fetal Stomach Content	2023

**Table 2:** **Gene targets and primer** **sequences used in this study.**

Gene	Primer Name	Sequence	Product Size	Reference
marA	marA-F	5′-TAGGCCAATACATCCGCAGC-3′	193	This study
	marA-R	5′-TACCGTGATTCGCCATGC-3′		
ramA	ramA-F	5′-CGCTCAGGTTATCGACAC-3′	179	This study
	ramA-R	5′-CCGCCAGTTTTAGCTTCC-3′		
acrA	acrA-F	5′-ACGACAAACAGGACCAGC-3′	161	This study
	acrB-R	5′-ACGCTTCAGGATAATGCC-3′		
acrB	acrB-F	5′-TCGTGTTCCTGGTGATGTACCT-3′	69	[21]
	acrB-R	5′-AACCGCAATAGTCGGAATCAA-3′		
soxS	soxS-F	5′-CGGAATACACGCGAGAAGGT-3′	72	[22]
	soxS-R	5′-GAGCGCCCGATTTTTGATATC-3′		
16S rRNA	16S-F 16S-R	5′-CGGGGAGGAAGGTGTTGTG-3′ 5′-GAGCCCGGGGATTTCACATC-3′	178	[23]

**Table 3:** **The minimum inhibition concentration** **(MIC) of the various isolates obtained of Ciprofloxacin.**

Number	Serovar	Accession	MIC (μg/mL) Disk Diffusion (μg/mL)	Interpretation	Selected for Gene Expression	MIC (μg/mL) Broth Dilution (μg/mL)
1	*Salmonella* Dublin	85-1235	<0.125	Susceptible	Yes	0.03
2	*Salmonella* Typhimurium	02-742	<0.125	Susceptible	No	-
3	*Salmonella* Enteritidis	09-10043	<0.125	Susceptible	No	-
4	*Salmonella* Typhimurium	13-427	<0.125	Susceptible	No	-
5	*Salmonella* Typhimurium	15-3130	<0.125	Susceptible	No	-
6	*Salmonella* Typhimurium	20-04335	<0.125	Susceptible	No	-
7	*Salmonella* Typhimurium	21-02381	<0.125	Susceptible	No	-
8	*Salmonella* Typhimurium	21-02399-B	<0.125	Susceptible	No	-
9	*Salmonella* Enteritidis	15V01330	<0.125	Susceptible	No	-
10	*Salmonella* Typhimurium	C202670019	<0.125	Susceptible	No	-
11	*Salmonella* Typhimurium	C210050040	<0.125	Susceptible	No	-
12	*Salmonella* Typhimurium	C210080050A	<0.125	Susceptible	No	-
13	*Salmonella* Typhimurium	C210460048-1	<0.125	Susceptible	No	-
14	*Salmonella* Typhimurium	C220050014	<0.125	Susceptible	No	-
15	*Salmonella* Typhimurium	C2301000070-2	<0.125	Susceptible	No	-
16	*Salmonella* Typhimurium	C230200031	<0.125	Susceptible	No	-
17	*Salmonella* Dublin	C230310056	1.500	Resistant	Yes	1.95

## Data Availability

The original contributions presented in this study are included in the article/Supplementary Material. Further inquiries can be directed to the corresponding authors.

## Supplementary Materials

The following supporting information can be downloaded at: https://www.mdpi.com/article/10.3390/vetsci13020177/s1, Figure S1: Serial dilutions of Ciprofloxacin prepared, showing the Minimum Inhibition Concentrations for both Ciprofloxacin-resistant and Ciprofloxacin-susceptible isolates of *Salmonella* Dublin and the respective concentrations used for gene-expression analysis; Supplementary Data—Table S1: Nucleotide Mutations identified in the Ciprofloxacin-resistant *Salmonella* Dublin isolate; Table S2: Antimicrobial resistance genes identified in the Ciprofloxacin-susceptible *Salmonella* Dublin isolate; Table S3: Antimicrobial resistance genes identified in the Ciprofloxacin-resistant *Salmonella* Dublin isolate; Table S4: Plasmids identified in the Ciprofloxacin-susceptible *Salmonella* Dublin isolate; Table S5: Plasmids identified in the Ciprofloxacin-susceptible *Salmonella* Dublin isolate.
