# Supplementary material for: Gene Expression Analysis and Whole Genome Sequencing Reveal the Potential Mechanism of Ciprofloxacin Resistance in a Salmonella Dublin Isolate
Source: Vet Sci. 2026 Feb 10;13(2):177. doi: 10.3390/vetsci13020177 (PMC12944894; doi:10.3390/vetsci13020177)
Supplement: Supplementary file 1 [file vetsci-13-00177-s001.zip › Figure S1.pptx]

## Slide 1
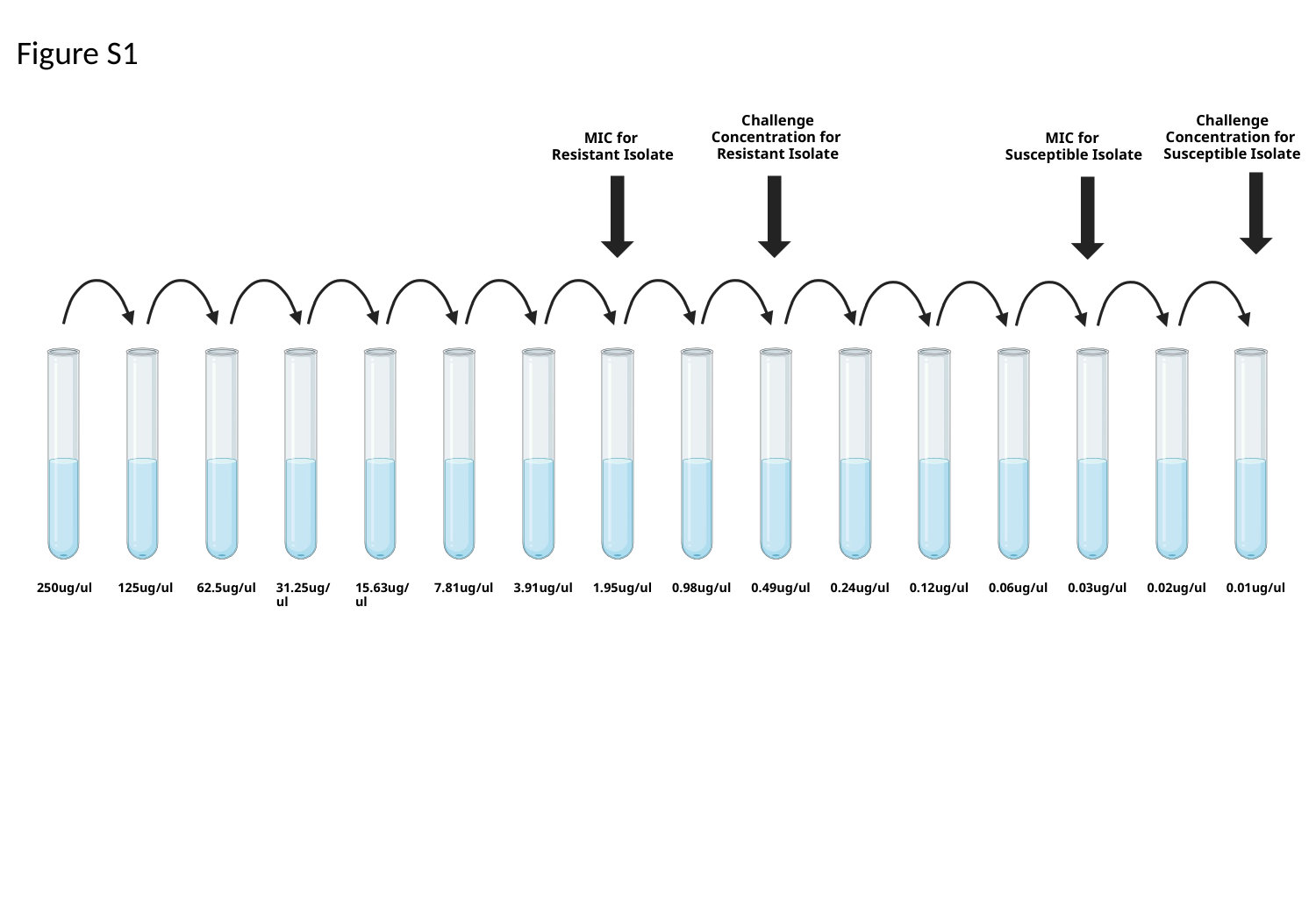

Figure S1
Challenge Concentration for
Susceptible Isolate
Challenge Concentration for
Resistant Isolate
MIC for
 Susceptible Isolate
MIC for
 Resistant Isolate
250ug/ul
125ug/ul
62.5ug/ul
31.25ug/ul
15.63ug/ul
7.81ug/ul
3.91ug/ul
1.95ug/ul
0.98ug/ul
0.49ug/ul
0.24ug/ul
0.12ug/ul
0.06ug/ul
0.03ug/ul
0.02ug/ul
0.01ug/ul
